# CSF neurofilament and N-acetylaspartate related brain changes in clinically isolated syndrome

**DOI:** 10.1177/1352458512458010

**Published:** 2013-04

**Authors:** M Khalil, C Enzinger, C Langkammer, S Ropele, A Mader, A Trentini, MLG Vane, M Wallner-Blazek, G Bachmaier, J-J Archelos, MJA Koel-Simmelink, MA Blankenstein, S Fuchs, F Fazekas, CE Teunissen

**Affiliations:** 1 Department of Neurology, Medical University of Graz, Graz, Austria; 2 Department of Clinical Chemistry, Neurological Laboratory, BioMS-eu, VU University Medical Center Amsterdam, Amsterdam, The Netherlands; 3 Department of Radiology (Division of Neuroradiology), Medical University of Graz, Graz, Austria; 4 Institute for Medical Informatics, Statistics and Documentation, Medical University of Graz, Graz, Austria

**Keywords:** Multiple sclerosis, MRI, brain atrophy, CSF, biomarker, axonal damage, neurofilament, N-acetylaspartate

## Abstract

**Background:**

Axonal damage is considered a major cause of disability in multiple sclerosis (MS) and may start early in the disease. Specific biomarkers for this process are of great interest.

**Objective:**

To study if cerebrospinal fluid (CSF) biomarkers for axonal damage reflect and predict disease progression already in the earliest stages of the disease, that is, in clinically isolated syndrome (CIS).

**Methods:**

We assessed CSF levels of neurofilament heavy (NFH), neurofilament light (NFL) and N-acetylaspartate (NAA) in 67 patients with CIS and 18 controls with neuropsychiatric diseases of non-inflammatory aetiology (NC). Patients with CIS underwent baseline magnetic resonance imaging (MRI) at 3T, and a follow-up MRI after 1 year was obtained in 28 of them.

**Results:**

Compared with NC, patients with CIS had higher NFH (*p*=0.05) and NFL (*p*<0.001) levels. No significant group differences were found for NAA. Patients’ NFH levels correlated with physical disability (*r*=0.304, *p*<0.05) and with change in brain volume over 1 year of follow-up (*r*=-0.518, *p*<0.01) but not with change in T2 lesion load.

**Conclusion:**

Our results confirm increased neurofilament levels already in CIS being related to the level of physical disability. The association of NFH levels with brain volume but not lesion volume changes supports the association of these markers with axonal damage.

## Introduction

Converging evidence suggests that axonal damage and neurodegeneration occur already in early phases of multiple sclerosis (MS).^1,2^ The extent of axonal damage and neurodegeneration may be driven by inflammatory processes^3^ and reflect a major determinant of patients’ physical disability.^4,5^

Assessment of cerebrospinal fluid (CSF), neurofilament (NF) and N-acetylaspartate (NAA) provide promising tools to indicate the degree of axonal damage.^6,7^ The presence of validated assays for NF heavy chain protein,^8^ NF light chain protein^9^ and NAA^10^ may help to pave the way for their clinical application.

While two recent studies provide evidence that CSF NF levels are altered already in early phases of the disease,^6,11^ studies on CSF NAA suggest that this may rather be a marker for axonal damage in progressive forms of MS.^6^ NF heavy levels have been shown to correlate with relapses and disability in MS.^6,11^

No information exists so far as to whether markers of axonal damage and neurodegeneration are related to morphological brain changes as measured by 3T MRI at the earliest clinical stage of MS, that is, in clinically isolated syndrome (CIS). Moreover, it is also unclear if these markers are predictive of the evolution of subsequent brain tissue damage. With this study we therefore aimed to confirm previous findings on CSF levels of NF light and heavy chain proteins and NAA in CIS and to explore if they could also serve as predictive markers for disease progression in CIS, that is, to analyse if their concentrations might indeed predict accumulating tissue damage as indicated by MRI.

## Subjects/materials and methods

### Patients and controls

From 2003 to 2010, we recruited 67 consecutive patients with CIS suggestive of MS^12^ from our MS outpatient clinic at the Medical University of Graz (Table 1).

**Table 1.** Demographical and clinical data.

	CIS	Controls	*p*-value
N (% female)	67 (70.1)	18 (66.7)	ns
Age at LP (years)^a^	33.4 (9.9)	34.7 (13.9)	ns
Age at disease onset (years)^a^	33.3 (9.9)	NA	NA
Disease duration at LP (months)^b^	0.3 (0.2–1.3)	NA	NA
EDSS at LP in remission^b^	1.0 (0.0–2.0)	NA	NA
N (% female) with clinical FU	46 (69.6)	NA	NA
Clinical FU (years)^b^	2.3 (1.4–3.4)	NA	NA
EDSS at FU in remission^b^	1.0 (0.0–2.0)	NA	NA
N (%) patients converted to MS during FU	17 (37.0)	NA	NA
N (% female) with follow-up MRI	28 (64.3)	NA	NA
MRI FU (years)^b^	1.0 (1.0–1.1)	NA	NA

CIS: clinically isolated syndrome, EDSS: Expanded Disability Status Scale, FU: follow-up, LP: lumbar puncture, MRI: magnetic resonance imaging, N: number of patients/controls, NA: not applicable, ns: not significant, MS: multiple sclerosis.

Values are presented as number (%), ^a^mean (SD) or ^b^median (interquartile range).

Patients underwent diagnostic lumbar puncture for CSF analysis, detailed clinical examination and a 3T MRI. Assessment of demographical and clinical data included age, age at disease onset, disease duration, Expanded Disability Status Scale (EDSS) score,^13^ occurrence of relapses and MS therapy. A relapse was defined as the appearance or reappearance of at least one neurological symptom or the worsening of an old symptom attributed to MS that lasted for at least 24 hours and which was preceded by a relatively stable or improving neurological state of at least 30 days. Clinical follow-up data were available in 46 patients with CIS (Table 1). None of the patients with CIS had received any MS-specific therapy at the time of the lumbar puncture.

We included 18 patients with other neuropsychiatric diseases of non-inflammatory aetiology and a normal CSF as controls (Table 1). This group consisted of 10 patients with headache, two with facial palsy, one with depression, one with pain, one with polyneuropathy, one with vertigo, one with peripheral nerve palsy and one patient with subjective complaints of sensory disturbances. CIS and control patients had similar mean age and gender distribution (Table 1).

### Standard protocol approvals, registrations and patient consent

The study was approved by the ethics committee of the Medical University of Graz. All participants gave written informed consent.

### Cerebrospinal fluid analyses

A total volume of 6–10 ml of CSF was obtained by lumbar puncture. After diagnostic work-up, excess of CSF was stored at -80°C until use in agreement with international consensus guidelines.^14^ All CSF analyses were performed by trained technicians or biochemists blinded to clinical information. In 46 patients (68.7%) lumbar puncture was performed during their first clinical attack, in 21 patients (31.3%) CSF was obtained during remission after their first attack. Remission was defined as a time lag of at least 14 days between the clinical attack and CSF withdrawal in conjunction with clinical improvement as documented in our patient files.

Neurofilament heavy (NFH) concentrations were assessed in 67 patients with CIS and 18 controls by an in-house developed multiplex assay as described previously.^15^ All analyses were performed in duplicate and normalized. Inter-assay coefficient of variance (CV) was 17.9 per cent and intra-assay CV was 3.9 per cent. The hook effect^16^ caused by NF aggregates was avoided by further dilution of the samples to ensure accurate quantification of the immunoassay.

Neurofilament light (NFL) concentrations were measured using a commercial available ELISA kit from Uman-Diagnostics AB (www.umandiagnostics.com). This ELISA kit has recently been validated in a multicentre study, which identified critical factors such as accurate standard preparation.^17^ Due to depletion of some CSF samples, NFL levels were obtained from 47 patients with CIS and 15 controls.

NAA was determined in 67 patients with CIS and 18 controls by stable isotope dilution gas chromatography-mass spectrometry method.^6,10^

### Magnetic resonance imaging

Patients underwent MRI at 3 Tesla (Siemens Tim Trio, Siemens Healthcare, Erlangen, Germany) using a phased-array head coil with 12 receiver elements and a consistent imaging protocol as described previously.^18,19^ There was a median time interval of 3.2 (IQR 1.0–6.2) months between the lumbar puncture and brain MRI at 3T. Follow-up MRI at the same magnet using identical protocols after 1 year was obtained from 28 patients with CIS (Table 1).

All image analyses were performed by trained and experienced technicians and MR readers, blinded to clinical information.

Brain tissue volume and hyperintense T2-lesion load were measured as described previously.^18,19^ In addition, separate estimates of volumes of grey matter, white matter, cortical grey matter and ventricular CSF, normalized for subject head size, were estimated using SIENAX, which is part of FSL.^20^ For assessing T2 lesion load, masks defining the lesions were created and the total lesion load was calculated by multiplying the area of all masks by the slice thickness.

### Statistics

Statistical analyses were performed using SPSS 18.0 (SPSS Inc., Chicago, Illinois, USA). The Kolmogorov–Smirnov test assessed normality of data distribution. Groups were compared by Mann–Whitney *U* test. Student’s t test was applied to compare mean age values between groups. Spearman correlations served to calculate the correlation coefficients between CSF, imaging and clinical data. Pearson partial correlations were performed on ranked variables to correct for age.

## Results

### Group differences of CSF NF and NAA levels and correlations with demographical data

**Figures 1A-C.**
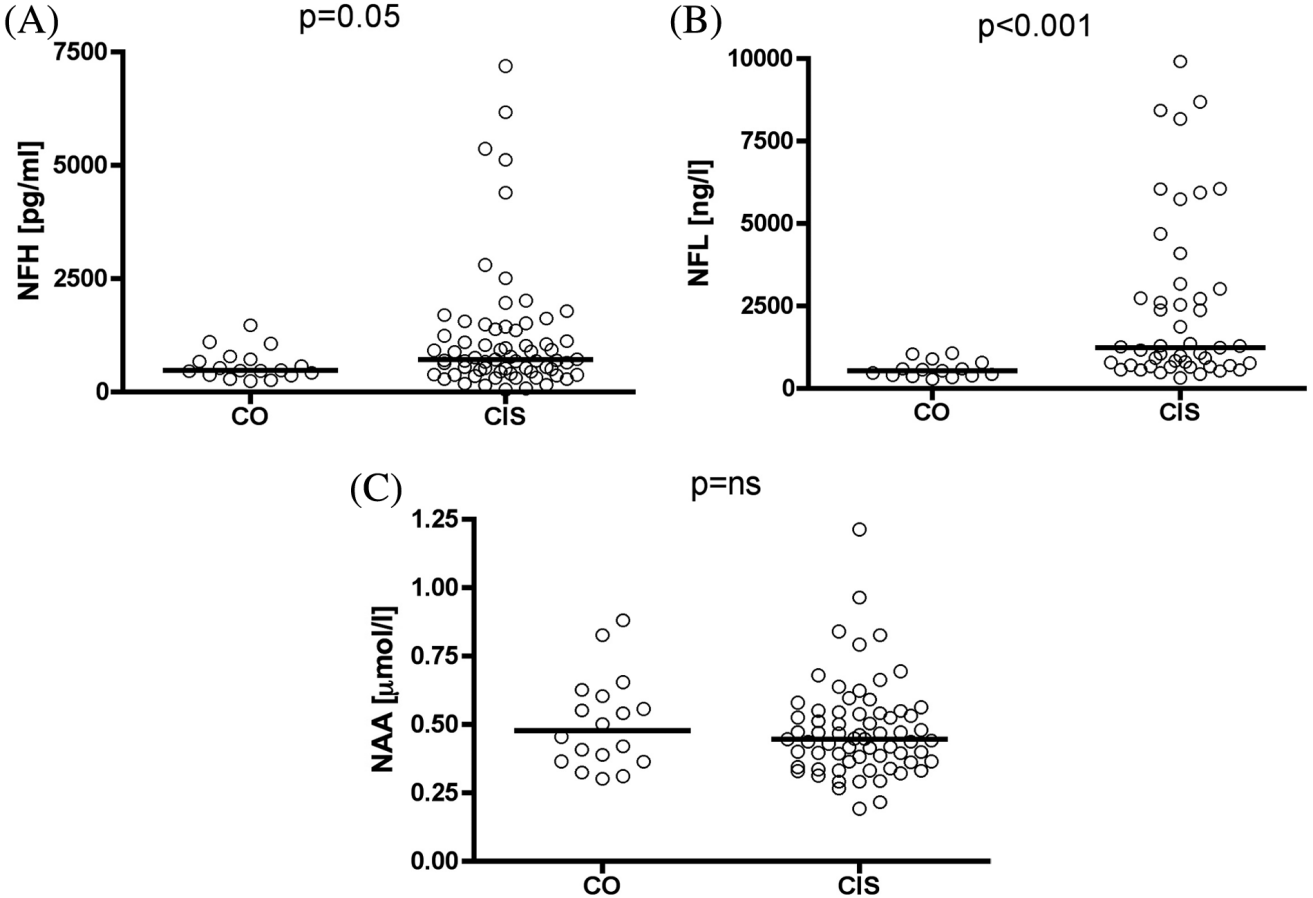
Group differences of CSF NFH, NFL and NAA between patients with CIS and controls. Patients with CIS had higher levels of NFH (A) and NFL (B). No significant group difference was observed regarding NAA levels (C). CIS: clinically isolated syndrome, CO: control, CSF: cerebrospinal fluid, NAA: N-acetylaspartate; NFH: neurofilament heavy, NFL: neurofilament light, ns: not significant.

Median levels of CSF of NFH and NFL were 1.5 fold and 2.3 fold higher, respectively, in patients with CIS compared with controls. NAA levels were not significantly different (Figures 1A–C). CSF NFH, NFL and NAA levels did not significantly differ between patients in remission compared with those with an acute relapse at the time of lumbar puncture (data not shown).

In controls, both NFH (*r*=0.713, *p*<0.005) and NFL (*r*=0.929, *p*<0.001) levels correlated with age. In patients with CIS, this correlation was less strong for NFH (*r*=0.325, *p*<0.01) and absent for NFL levels (*r*=0.145, *p*>0.05). NAA levels did not correlate with age at time of lumbar puncture.

### Correlations of CSF NF and NAA levels with clinical data

EDSS score at initial assessment at clinical stability after the first attack positively correlated with NFH (*r*=0.362, *p*<0.01), NFL (*r*=0.324, *p*<0.05) and NAA levels (*r*=0.284, *p*<0.05). After adjusting for age, this correlation remained significant for NFH (r=0.304, p<0.05; data adjusted for age) and NAA (*r*=0.308, *p*<0.05; data adjusted for age).

CSF NFH, NFL and NAA levels were not significantly different in patients with CIS who later on converted to MS compared with non-converters (data not shown).

### Correlations of CSF NF and NAA levels with MRI parameters

**Figure 2.**
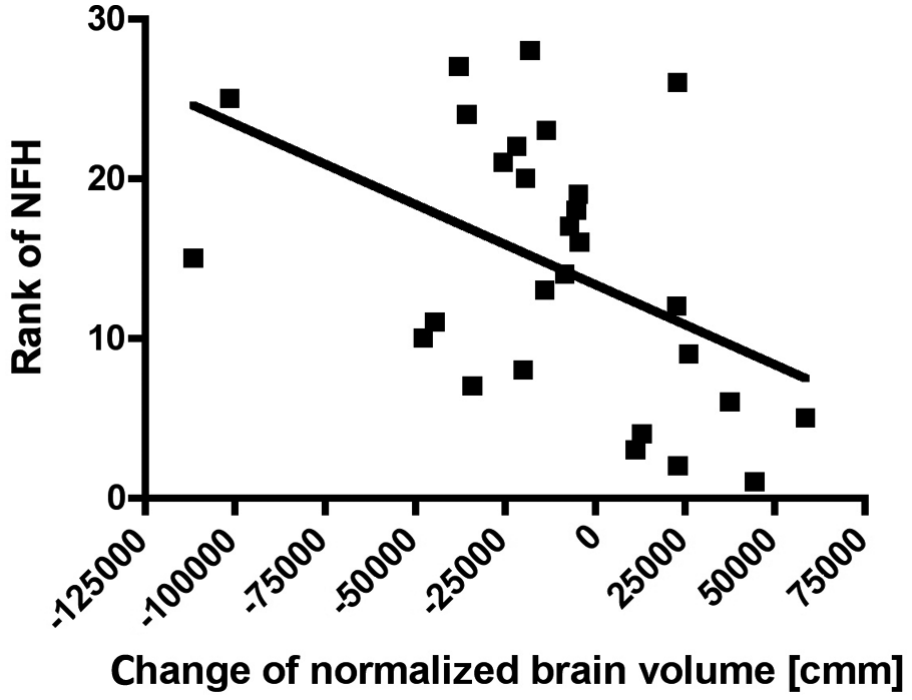
Correlation of CSF NFH levels (ranked variable) with the change of normalized brain tissue volume over time. Higher NFH levels are associated with increased brain tissue loss over time (*r*=-0.518, *p*<0.01; Pearson partial correlation corrected for age). CSF: cerebrospinal fluid, NFH: neurofilament heavy.

There were no significant correlations of NFH, NFL and NAA levels with baseline normalized volumes of whole brain, grey matter, white matter, cortical grey matter and ventricular CSF (adjusted for age). Higher CSF NFH levels correlated with accelerated global brain volume decrease over a median follow-up of 1.0 years (*r*=-0.518, *p*<0.01; data adjusted for age) (Figure 2). There were no significant correlations between NFH, NFL or NAA levels neither with baseline T2 lesion volume, nor with its change over time during follow-up.

### Correlations among CSF NF and NAA levels and relation to other CSF parameters

NFH levels correlated with NFL levels in the total group of patients (*r*=0.551, *p*<0.001; data adjusted for age). NAA levels did not correlate with NFH and NFL levels.

**Figure 3.**
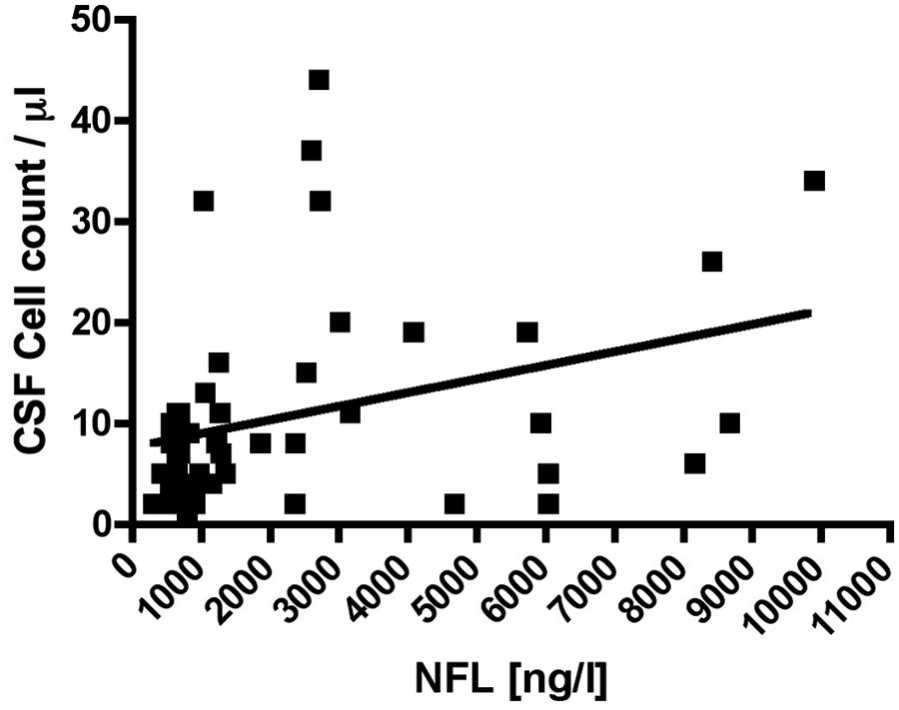
Scatter plot of CSF NFL levels and CSF white cell count variables (unranked and not corrected for age). NFL levels were significantly correlated with white cell count (*r*=0.646, *p*<0.001, Pearson partial correlation corrected for age). CSF: cerebrospinal fluid, NFL: neurofilament light.

Routine CSF parameters are listed in Table 2. NFH (*r*=0.337, *p*<0.005, data adjusted for age) and NFL (*r*=0.646, *p*<0.001, data adjusted for age) levels significantly correlated with CSF cell count. Figure 3 represents the correlation of NFL levels with CSF cell count. Both NFH (*r*=0.252, *p*<0.05, data adjusted for age) and NFL (*r*=0.456, *p*<0.001, data adjusted for age) levels correlated with the IgG index and NFL levels correlated with albumin quotient (*r*=0.348, *p*<0.01, data adjusted for age). No significant correlations between NAA and IgG index or albumin quotient were present.

**Table 2.** CSF parameters in patients with CIS and controls.

	CIS	Controls	*p*-value
N	67	18	
Cell count, N/µl (Ref. ≤4)	8.0 (4.0–14.0)	1.5 (1.0–2.0)	<0.001
CSF protein, mg/dl	34.0 (29.0–44.0)	28 (22.0–34.0)	<0.01
Q_alb_	5.0 (4.2–7.0)	3.8 (3.4–5.5)	<0.05
IgG index	0.8 (0.6–1.2)	0.5 (0.5–0.5)	<0.001
OCB positive, N (%)	63 (94.0)	ND	NA

CIS: clinically isolated syndrome, CSF: cerebrospinal fluid, N: number, NA: not applicable, ND: not done, OCBs: oligoclonal bands, Q_alb_: albumin quotient.

Values are given as number, number (%) or as median (interquartile range).

## Discussion

The association of NFH levels with subsequent brain tissue damage over a period of 1 year as defined by MRI is a main and new finding. Our study confirms that CSF NFH and NFL levels are already increased in patients with a CIS suggestive of MS.^6,11^ This preliminary observation suggests that CSF NFH protein levels may predict progressive brain damage specifically caused by axonal damage.

The relationship between CSF NF and 3T MRI measures of tissue damage in CIS has not been investigated so far. One study reported a correlation between the CSF/serum index of antibodies directed against NFL and brain atrophy, but this study analysed only patients with relapsing remitting or progressive forms of MS, and no relation with NF proteins was observed.^21^ None of the CSF markers of axonal damage analysed in our study correlated with T2 lesion volume at baseline or its change over time. This partly contrasts a previous finding where a correlation of NFL levels with the number of T2 lesions has been described in a combined analysis of patients with CIS or MS.^6^ Another study found an association of CSF NFH levels with the volume of enhancing lesions in patients with MS.^22^ However, in our CIS cohort, the prevalence of enhancing lesions was too low to allow such an analysis.

Our findings of increased CSF NFH and NFL levels in CIS support earlier suggestions^6,11^ and argues for axonal damage to occur already in the earliest stage of the disease, which is also corroborated by recent neuropathological findings.^1^ NFs are the major axonal cytoskeletal proteins.^7^ After axonal injury they are released into the extracellular compartment, and subsequently into the CSF and peripheral blood.^23^ Thus analysis of CSF/serum NF levels may provide a valuable tool to estimate the extent of axonal damage in patients with MS.^24^ Apart from measuring NF levels in CSF a few reports have also suggested NFH in peripheral blood as a potential marker of neurodegeneration in MS^25,26^ but no reports are yet available regarding NFL. Thus further studies on NF in peripheral blood are needed before recommending these tests for assessing MS prognosis. Potential clinical applications of assessment of neuroaxonal damage markers have been illustrated by two recent studies showing that NF levels may indicate treatment efficacy with the monoclonal antibody natalizumab,^27^ and further capture neurotoxic side effects of aggressive treatment regimens.^28^

Another neuroaxonal marker is NAA.^7^ Quantification of NAA in the extracellular fluid of the CNS was first described in patients with traumatic brain injury using cerebral microdialysis.^29^ Evidence for a clinical relevance of NAA comes from several studies, showing that both higher serum and CSF NAA levels correlated with physical disability in relapsing–remitting patients with MS.^10,30^ Compared with controls, CSF NAA levels were not altered in CIS in the current study, supporting previous findings of decreased NAA levels rather in later stages of the diseases such as in secondary progressive MS.^6^

In the control group, we observed a close correlation of NFH and NFL levels with age. This correlation was less strong in CIS for NFH and absent for NFL levels. In line with a recent report on NFH,^11^ these findings suggest that age-related changes in the levels of CSF NF may at least partly be caused by ongoing neurodegeneration, which increases with age. In contrast, this age effect may be covered by pathological conditions, where increased brain tissue damage leads to higher CSF NF levels. Due to the correlation of NFs with age, statistical analyses require correction for this variable to ensure adequate clinical interpretation.^11^

In this respect one should also mention that compared with our luminex-based NFH assay, another electrochemiluminescence immunoassay for NFH assessment with higher sensitivity has recently been developed.^8^ In order to compare both assays direct comparison studies need to be performed, exchanging protein standards, using the same quality controls (low and high) and measuring the same low- concentration CSF samples in every assay.

After adjusting for age, NFH and NAA levels were correlated with EDSS in remission after the first attack, but the correlation was not very strong. Although NFH levels have been shown to correlate with EDSS also by several other groups,^6,11,22,28,31^ our results still need to be regarded with caution since the range of the EDSS score in our CIS cohort was relatively small. Previous results on correlations of NFL with EDSS^6,32^ could not be confirmed by our study when correcting for age.

Although we did not observe any group differences for NAA, higher NAA levels correlated with the EDSS score. This argues for a temporal dynamic of NAA levels as suggested previously.^6^ Whereas in early disease phases CSF NAA levels increase upon axonal damage, decreased levels are observed in advanced stages of the disease.^6,10^

When comparing levels of NF and NAA in patients who converted to MS during follow-up compared with non- converters, no significant differences in biomarker levels emerged. Thus, we could not confirm previous reports on altered NFH, NFL or NAA levels in patients with CIS who converted to definite MS compared with non-converters.^6,33^ In our CIS cohort, we could also not find any differences for NFH, NFL and NAA comparing patients whose CSF was obtained in remission compared with acute patients, as reported previously.^6,11,32^

Another interesting finding of our study is that both NFH and NFL but not NAA levels correlated with CSF white cell count. Such association has been reported previously for NFL^32^ but not for NFH^11^ and no reports are yet available regarding NAA.^6,10^ Our findings suggest that NFH and NFL may thus be rather related to inflammatory disease activity.^22,34^ The correlation of NFH and NFL with the IgG index further supports this notion, but this correlation has not been found in a recently published study.^11^ We found a correlation of the albumin quotient only with NFL levels, which has not been described in other studies on CSF NFL chain protein.^9,32^ In line with previous reports no such relation was found for NFH levels^11^ and the NFH and NFL levels were correlated.^6^

Our results indicate that analysis of both NF subunits in the CSF markers is relevant in CIS since their relation to clinical and paraclinical findings are different. Whereas NFL more than NFH is closer related to inflammatory signs such as CSF white cell count and IgG index, NFH rather indicates permanent axonal damage and neuronal degeneration which is documented by its relation to physical disability and brain volume loss over time already over the short term. Thus, assessment of CSF NFs in CIS may be of clinical importance to help stratifying patients with more aggressive disease already at the earliest stage of the disease. Although there is good evidence for CSF NAA to serve as a marker for axonal damage in established and progressive MS,^6^ our results indicate that it is of minor importance in patients with CIS.
